# Get to Know the National Institute on Aging: NIA Session for Early-Career Researchers

**DOI:** 10.1093/geroni/igaf122.1147

**Published:** 2025-12-31

**Authors:** Kenneth Santora, Kenneth Santora

## Abstract

The National Institute on Aging (NIA) at the National Institutes of Health, Department of Health and Human Services, supports biomedical and behavioral research with a lifespan focus. NIA research seeks to understand the basic processes of aging, improve prevention and treatment of diseases in later life, and improve the health of older persons, in addition to an emphasis on Alzheimer’s disease and related dementias. NIA supports a variety of training and career development opportunities for early-career investigators, including undergraduates, advanced degree students, post-docs, and junior faculty. In this session, we will provide an overview of NIA-funded research, followed by a presentation on funding mechanisms and strategies to consider when applying for extramural grants. Finally, attendees will be able to join any number of breakout group discussions led by extramural NIA training staff and program officials to discuss opportunities and resources specific to various career paths.

